# Bifunctional Silica-Supported Ionic Liquid Phase (SILP) Catalysts in Silane Production: Their Synthesis, Characterization and Catalytic Activity

**DOI:** 10.3390/ijms25010068

**Published:** 2023-12-20

**Authors:** Nataliia V. Abarbanel, Sergey S. Suvorov, Anton N. Petukhov, Artem S. Belousov, Artem N. Markov, Dmitriy M. Zarubin, Alexandra V. Barysheva, Ilya V. Vorotyntsev, Alexander A. Kapinos, Artem D. Kulikov, Andrey V. Vorotyntsev

**Affiliations:** 1 Chemical Engineering Laboratory, Lobachevsky State University of Nizhny Novgorod, Gagarina Avenue 23, Nizhny Novgorod 603950, Russiabelousov@ichem.unn.ru (A.S.B.); markov.art.nik@gmail.com (A.N.M.); dimazarubin493@gmail.com (D.M.Z.); alex.barysheva@yandex.ru (A.V.B.); an.vorotyntsev@gmail.com (A.V.V.); 2 Laboratory of Smart Materials and Technologies, Mendeleev University of Chemical Technology of Russia, 9 Miusskaya Square, Moscow 125047, Russia

**Keywords:** SILP, imidazole, heterogeneous catalysis, trichlorosilane, disproportionation, silane

## Abstract

A mesoporous silica support was synthesized using the sol–gel method from trichlorosilane. There is a tendency for the specific surface area and the proportion of silica particles mesopores to increase during all stages of sol–gel synthesis. It has been shown that the insertion of hexane and toluene, as additional solvents, into the structure-forming polyethylene glycol, makes it possible to regulate the pore size and specific surface area of silica. Silica functionalization was carried out using SILP technology. The activities of the catalytic systems based on polymer and inorganic supports immobilized by imidazole-based ionic liquids during the trichlorosilane disproportionation reaction were compared. There is a tendency for the monosilane yield for catalytic systems based on an inorganic support to increase. We identified the most promising catalyst in terms of monosilane yield and proposed a bifunctional catalyst that exhibited activity in two parallel reactions: trichlorosilane disproportionation and silicon tetrachloride hydrogenation.

## 1. Introduction

The rapid development of industry has been associated with an increase in electricity consumption, which has increased the burden on the environment due to the need to increase the extraction and processing of organic energy resources. Despite a number of problems (high capital costs and dependence on the state of the environment), solar energy systems are gaining in popularity due to their ability to generate clean and renewable energy [1,2,3]. The main raw material for solar panels’ manufacture is polycrystalline silicon. In addition, the need for high-purity polycrystalline silicon has increased with the development of the micro- and nanoelectronic industries. Two industrial processes for polysilicon production can be distinguished: the Siemens process and the Union Carbide process. The first is based on the hydrochlorination of technical silicon into trichlorosilane, followed by its separation from the vapor–gas mixture and hydrogen reduction to polysilicon [4,5]. The Siemens process is quite energy-intensive (the temperature of the SiHCl_3_ hydrogen reduction is 1200 °C) and highly toxic; SiCl_4_ (in an amount of 18 tons per 1 ton of the main product) and HCl are released as by-products [6,7,8]. In the Union Carbide process, monosilane (a precursor of polycrystalline silicon) is obtained as the result of the catalytic disproportionation of trichlorosilane (process temperature—80 °C), shown by reaction Equations (1)–(3) [9]. The advantages of this method are its low energy consumption due to the lower process temperatures (the temperature for the pyrolysis of silane in a fluidized bed is only 800 °C), and a lower level of environmental pollution. However, its significant disadvantage is the low practical yield of the main target product, SiH_4_—1.2%, compared to its theoretical yield of 5.9%. Thus, increasing the yield and selectivity of monosilane would reduce the cost of polycrystalline silicon and increase its industrial and environmental safety. The solution to the problem of trichlorosilane catalytic disproportionation technology is the search for effective catalytic systems.

(1) $$2{\mathrm{SiHCl}}_{3}\;⇄\;{\mathrm{SiCl}}_{4}+{\mathrm{SiH}}_{2}{\mathrm{Cl}}_{2}$$

(2) $$2{\mathrm{SiH}}_{2}{\mathrm{Cl}}_{2}\;⇄\;{\mathrm{SiCl}}_{3}+{\mathrm{SiH}}_{3}\mathrm{Cl}$$

(3) $$2{\mathrm{SiH}}_{3}\mathrm{Cl}\;⇄\;{\mathrm{SiH}}_{2}{\mathrm{Cl}}_{2}+{\mathrm{SiH}}_{4}$$

The authors of work [10] conducted a comparative analysis of three different silane-synthesis routes: using magnesium silicide, silicon halide and trichlorosilane catalytic disproportionation. They also concluded that the latter route is the most promising method for monosilane production, taking into account the economic and environmental factors. However, its industrial use is hampered by the lack of a catalyst that does not contaminate the product and can maintain its activity for a long time. Thus, solving the problem of the technology used for trichlorosilane catalytic disproportionation, in terms of searching for effective catalytic systems, is an urgent task. The most promising solution for this purpose is the use of catalysts based on ionic liquids (ILs). Research on the combination of ILs and solid supports (SILPs) has great potential, since their use allows for an increase in the reaction rate and selectivity, due to the similarity of SILP properties to those of molecular catalysts with homogeneous active centers [11,12,13]. It has been found [14,15,16] that functional groups containing nitrogen heteroatoms exhibit high catalytic activity in the trichlorosilane disproportionation reaction. It was previously shown [17] that imidazolium-based SILPs exhibit high catalytic activity in the Union Carbide process and, therefore, are of interest for further research, for example, in terms of replacing the organic porous support with a more chemically and thermally stable silica with a high specific surface area [18]. This is important because the determination of an optimal catalytic system is based not only on the properties of the IL functional group; other important factors here are the characteristics of the porous support: its specific surface area, average particle diameter, pore size distribution and surface morphology [19,20,21,22]. The most widely used silica particle production technology is the sol–gel method, since it allows for the obtainment of a pure material, with the ability to control the morphology, phase composition and particle size, in contrast to flame synthesis. The use of the template method is complicated by the need to use high-cost commercial micelle-forming agents (for example, Pluronic F127) [23,24,25,26]. In the sol–gel method, the more affordable and cost-effective polyethylene glycol (PEG) can be used as the structure-organizing agent [27,28,29,30,31,32,33]. It is a simple linear, synthetic polymer with varying molecular weights and viscosities. Due to its hydrophobic and hydrophilic segments, it is soluble in aqueous and organic solvents, and under certain conditions, it forms an oscillating network of macromolecules and flocculates silica particles. The precursors of SiO_2_ are hydrolyzable silicon compounds: the inorganic salts of sodium silicate (Na_2_SiO_3_) or alkoxysilanes; tetraethoxysilane (TEOS) has traditionally been used. To reduce the cost of the technology, it is advisable to consider silicon tetrachloride (STC) and trichlorosilane (TCS) as precursors since they have a lower cost compared to tetraethoxysilane (TEOS) and are obtained by the esterification of silicon tetrachloride with ethanol [34]. In [35], a comparison of two silica precursors (TEOS and STC) was presented, and the influence of various factors on the morphology and structural characteristics of the final product was analyzed. It has been shown that nanoporous silicon dioxide obtained from STC is characterized by a larger specific surface area and a higher concentration of silanol groups. In the present work, for the first time, another large-tonnage product of the chemical industry is considered as a precursor of SiO_2_—TCS. The functionalization of the obtained porous silicon dioxide was carried out using supported ionic liquid phase (SILP) technology. The activities of the catalytic systems, based on the inorganic and organic supports in the reaction of trichlorosilane disproportionation, were compared. In order to process the main by-product of the reaction, STC, the possibility of using a system with two types of active centers, which trigger two parallel reactions, was considered: the disproportionation of TCS and the hydrogenation of STC. In this work, for the first time, bifunctional catalysts based on SILPs and metal chlorides (rhenium, nickel and cobalt), which have shown good results in the STC hydrogen reduction reaction [36,37], are proposed.

## 2. Results

### 2.1. Sample Characteristics

The main stages of the sol–gel process using silicon compounds as a precursor are as follows: hydrolysis with the formation of silanol groups; condensation with the formation of siloxane bridges, responsible for the silica structure building; aging, in which the gel strength increases because of the particle aggregation and structure compaction; drying to remove liquid from the gel spatial structure; calcination, which ensures the stability of the silicas’ textural and structural properties. Each stage’s effect on the resulting nanostructure surface properties was analyzed by conducting low-temperature nitrogen adsorption at 77 K (Table 1). After each stage, the samples underwent washing, water and solvents were removed by heating to 60 °C under vacuum conditions, and surfactants were eliminated through annealing at 550 °C. Sample designation is as follows: Sx_HT_AHTx_Vx_Tx, where Sx is the sample number, HT is the hydrothermal treatment, AHTx is the temperature of the hydrothermal treatment in hydrochloric acid, Vx is the drying temperature under reduced pressure and Tx is the calcination temperature in the muffle furnace. 

**Figure 1:**
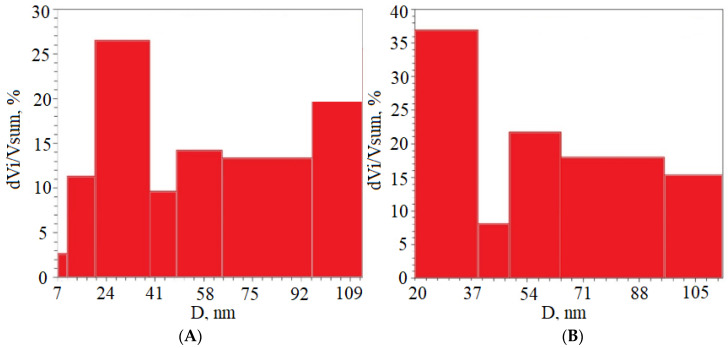
Pore size distribution relative to total volume (**A**–S5_HT_AHT100_V100_T550, **B**–S6_HT_AHT100_V100_T550).

**Figure 2:**
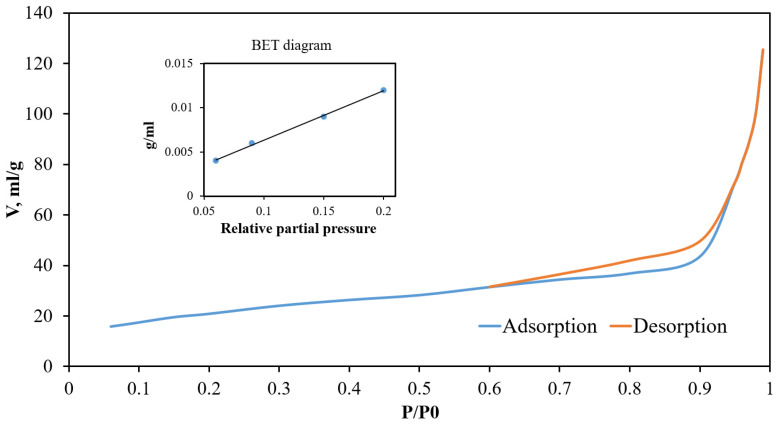
Nitrogen adsorption–desorption isotherms at 77 K for S6_HT_AHT100_V100_T550.

**Figure 3:**
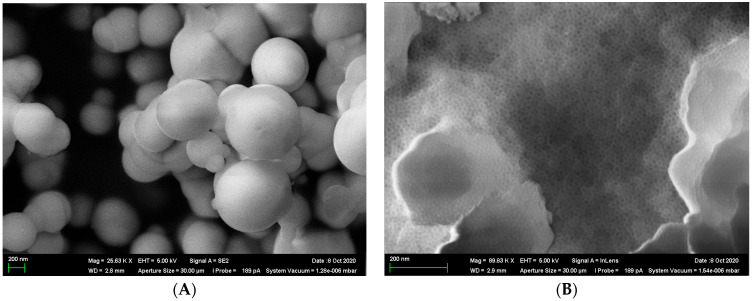
SEM images of SiO_2_ samples from TCS ((**A**) toluene; (**B**) hexane).

**Figure 4:**
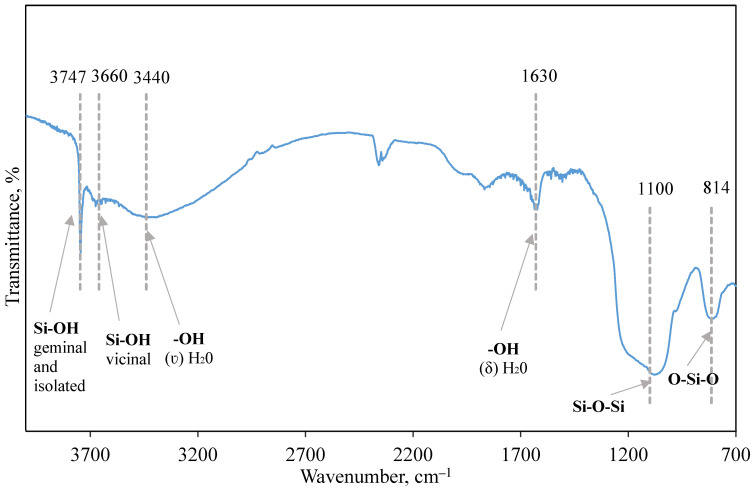
IR spectra of S6_HT_AHT100_V100_T550.

The pore size distribution relative to the total volume obtained using the BJH model is presented in Figure 1. Adsorption and desorption isotherms were constructed for the obtained samples. As an example, Figure 2 shows isotherms for the sample S6_HT_AHT100_V100 T550. Figure 3 shows SEM micrographs of the SiO_2_ samples from TCS. DRIFTS was used to evaluate the isolated silanol group content involved in functionalization in the S6_HT_AHT100_ V100 _T550 sample (Figure 4).

**Figure 5:**
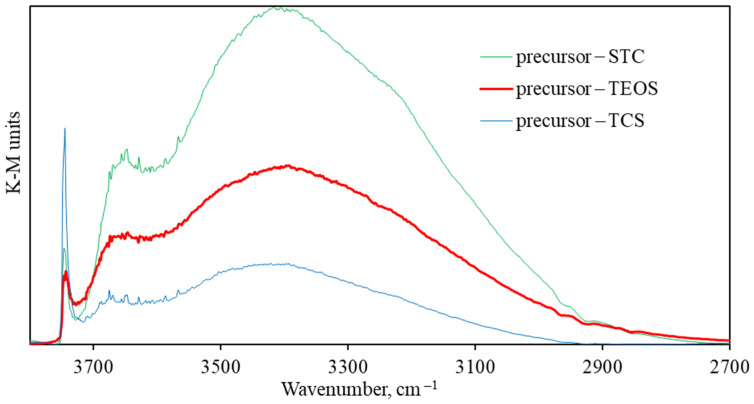
IR spectra of DRIFTS in K-M units of the samples synthesized from TEOS, STC and TCS.

The Kubelka–Munk theory adequately explains the dependence of the diffusely reflected light intensity and the medium optical characteristics. The intensity of adsorbate bands in the Kubelka–Munk (K-M) function units is directly related to the adsorbate molecules’ concentration. Figure 5 shows the IR spectra of DRIFTS in K-M units of the previously studied silica samples obtained from STC and TEOS [35], in comparison with the samples from TCS researched in this work.

**Figure 6:**
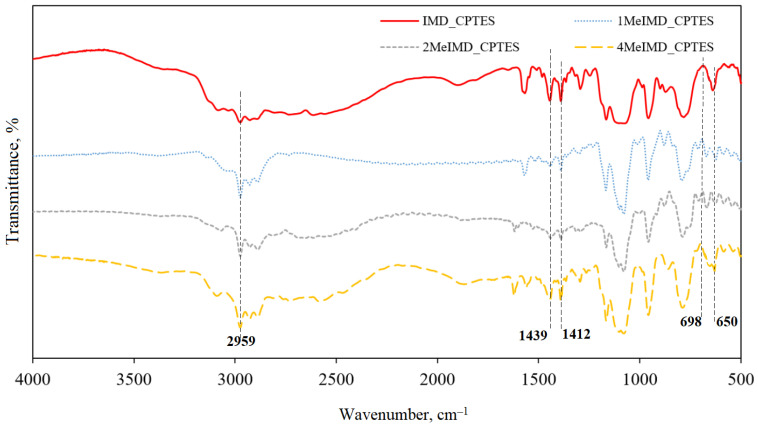
IR spectra of ionic liquids: IMD_CPTES; 1MeIMD_CPTES; 2MEIMD_CPTES; 4MeIMD_CPTES.

DRIFTS was used to evaluate the effectiveness of IL synthesis based on imidazole, 1-methylimidazole, 2-methylimidazole and 4-methylimidazole. The IR spectra of the resulting ILs are shown in Figure 6.

**Figure 7:**
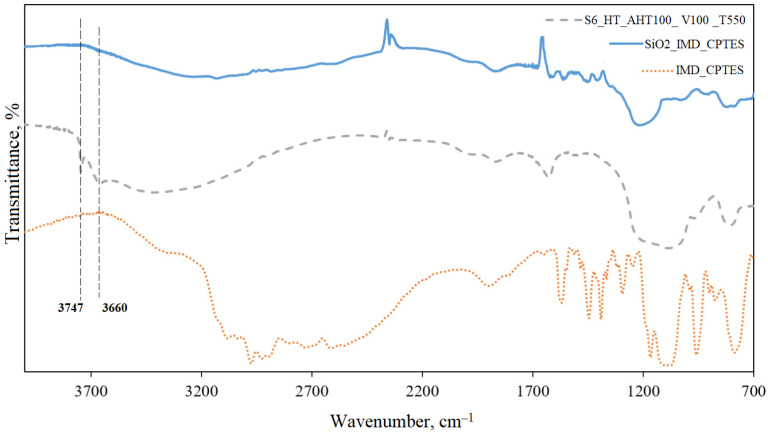
IR spectra of S6_HT_AHT100_V100_T550, IMD_CPTES and SiO_2__IMD_CPTES.

Next, the synthesized ILs were immobilized on the porous support, and DRIFTS was used to evaluate the immobilization success. Figure 7 shows the IR spectra of the S6_HT_AHT100_V100_T550 silica sample, the IMD_CPTES IL and the SiO_2_ sample after IMD_CPTES immobilization.

### 2.2. Comparison of Catalytic Activities

The catalytic systems were evaluated under static conditions to determine the system with the highest conversion and monosilane yield. Furthermore, the researched catalytic systems’ activities were compared with those of corresponding systems based on an organic support, which were obtained previously [13] under identical conditions (Table 2).

Then, a catalytic system containing two types of active centers was synthesized to trigger two parallel reactions: TCS disproportionation and STC hydrogenation. Catalysts based on rhenium, nickel and cobalt chlorides exhibited good results in the STC hydrogen reduction [36,37]. Therefore, in this research, the bifunctional catalysts’ activity based on transition metal chlorides was evaluated at a temperature of 160 °C. The DCS and STC yields were evaluated using chromatography–mass spectrometry through direct input into the mass spectrometer at *m*/*z* 99 (DCS molecular ion) and *m*/*z* 177 (STC molecular ion). GCMS was used to evaluate the impurity composition on a capillary column with a stationary phase based on trifluoropropylmethylpolysiloxane with a film thickness of 1.5 μm. Next, an additional reagent, hydrogen, was introduced, leading to the occurrence of the STC hydrogen reduction reaction alongside the TCS disproportionation. The results are presented in Table 3.

## 3. Discussion

### 3.1. Sample Characteristics

The addition of PEG during the sol–gel synthesis process promotes the porous structure formation of the resulting silica materials, which affects the nucleation rate and the aggregation degree of primary particles [31,32,33]. The microporous silica materials’ synthesis condition is the formation of a spatial fluctuation network of PEG macromolecules, which acts as a flocculant that promotes the formation of close-packed continuous aggregates. In addition, the rigid fluctuation macromolecule framework formed during the drying process prevents the collapse of micropores. PEG-1500 is a low-molecular-weight agent; the length of its macromolecules is not sufficient to bind particles into dense aggregates, which leads to mesopore formation. Mesoporous materials, when used as catalyst carriers for crucial industrial reactions, provide a balance between a large specific surface area and effective gas transport characteristics. As seen from Table 1, in samples S5 and S6, mesopores really predominate: for the sample with toluene—29 nm (27%); for the sample with hexane—29 nm (36%).

The tendency to increase the specific surface area and the silica particles’ mesopore proportion at all stages of sol–gel synthesis is associated with a change in the material dispersion because of the thermal mobility of silica particles and PEG polymer chains. During hydrothermal treatment, smaller silica particles with higher curvature dissolve, and the condensation of silicic acids takes place on the surface of large particles, resulting in their further growth. Consequently, larger particles fuse together, transforming the globular structure into a spongy one.

Toluene and hexane were added as supplementary PEG solvents in order to manage pore size and specific surface area. SEM micrographs of the samples (Figure 3) show that the addition of toluene resulted in the agglomerate formation of regular-shaped spheres, and the usage of hexane led to the creation of a cellular hexagonal structure. This explains the observed increase in the specific surface area of silica particles when hexane was used as a replacement for toluene (Table 1).

According to the IUPAC classification [38], the adsorption and desorption isotherm curve (Figure 2) can be classified as a type V isotherm, a type III isotherm variation that shows hysteresis caused by capillary condensation on mesoporous materials, aligning with the aforementioned results. This isotherm type shows weak adsorbate–adsorbent interactions; therefore, insignificant adsorption is observed at the initial site of the isotherm. Adsorption increases as the surface becomes filled with adsorbed molecules, as the adsorbate molecules have a stronger interaction with each other than with the adsorbent surface. The hysteresis can be classified as type C, which indicates wedge-shaped pores with open ends.

**Figure 8:**
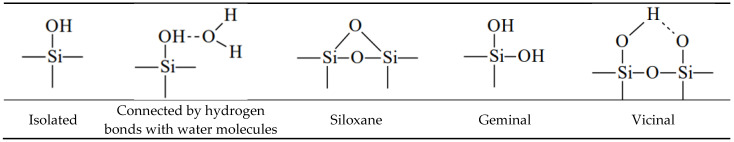
Different types of isolated silanol groups.

Since the immobilization of ionic liquids to the silica surface occurs through silanol groups, an important characteristic of such supports is their concentration. Silanol groups in silica are located both on the outer surface and in the material’s deeper layers. Surface groups, because of their accessibility, are important for IL immobilization. Their high activity is associated with their weak acid nature, due to which the proton easily enters exchange reactions. Besides isolated silanol groups, other group types can be present on the silica surface in different proportions (Figure 8) [39].

Figure 4 shows the IR spectrum of the S6_HT_AHT100_V100_T550 sample; isolated and geminal Si-OH groups are presented, and a peak is observed at 3747 cm^−1^. A peak in the region of 3660 cm^−1^ confirms the presence of vicinal Si-OH groups linked by hydrogen bonds. The wide band presence near 3440 cm^−1^ specifies -OH stretching vibrations. Water vibrations are observed in the area of 1630 cm^−1^. Stretching asymmetric vibrations of Si-O-Si and O-Si-O are observed at 1100 cm^−1^ and 814 cm^−1^, respectively. The obtained results lead to the conclusion that the condensation between Si-OR groups was successful.

According to the IR spectra of DRIFTS in K-M units (Figure 5), the samples where TCS was used as the initial precursor exhibited an isolated silanol group concentration of 0.11 K-M units, while with STC, it was 0.05 K-M, and with TEOS, it was 0.04 K-M. This indicates a high potential for using TCS for the production of silica supports.

Supported ILs have great potential as catalysts because they offer the ability to adjust the catalyst surface extensively through modification of the organic cation. This allows for the utilization of the versatility of ionic liquids in heterogeneous catalysis. As already mentioned, the SiO_2_ functionalization was provided using the SILP technology. For this, the synthesis of ILs based on imidazole, 1-methylimidazole, 2-methylimidazole and 4-methylimidazole was performed. Imidazolium-based SILPs, stabilized on divinylbenzene and chloromethylstyrene catalyst supports, were selected because of their high catalytic activity in the Union Carbide process [17]. DRIFTS was used to evaluate the synthesis effectiveness (Figure 6). The broad C-Cl bond band absence at 698 cm^−1^ due to the chlorine transition to the anion indicates the success of the IL synthesis. C-H vibrations can be observed at 2959 cm^−1^. The bands of about 1439 and 1412 cm^−1^ were attributed to the C-H vibrations of CH_2_ groups—symmetric and asymmetric vibrations. The absorption bands appearing at 650 cm^−1^ were attributed to the stretching of CH_2_-Si.

DRIFTS was used to evaluate the effectiveness of the immobilization of the synthesized ILs (Figure 7). The absence of silanol group peaks at 3747 cm^−1^ and 3660 cm^−1^ on the SiO_2__IMD_CPTES spectrum indicates that it completely covered the support surface with an organic cation.

### 3.2. Comparison of Catalytic Activities

The general trend observed in Table 2 in the series from imidazolium chloride to 4-methylimidazolium chloride is maintained. It was previously shown that the limiting stage of trichlorosilane disproportionation is the desorption of STC from the catalyst’s active sites. The calculated desorption activation energy values make it possible to explain the difference in catalytic activity and monosilane yield among the researched catalytic systems: the highest value is for PS/1MeIMD^+^Cl^−^, and the lowest is for PS/4MeIMD^+^Cl^−^ [17].

Catalytic systems based on an inorganic support demonstrate a tendency to increase the monosilane yield. This is attributed to these catalytic systems’ specific surface area exceeding the specific surface area of catalytic systems based on a polymer support by more than 6 times. Therefore, the number of active centers increases. However, as the experiment was conducted under conditions of a TCS deficiency on the catalytic site number, significant amounts of reactants and products were not desorbed and, accordingly, were not reflected in the results. This research has a qualitative nature, and its goal is to determine the most promising catalyst.

Nevertheless, the main point here is the catalytic activity tendency preservation, which indicates similar interaction mechanisms because of the nature of the catalytic centers. Another significant aspect is the potential use of catalytic systems with inorganic supports at higher temperatures, which contributes to an increase in the rate of STC desorption from the catalyst active sites, causing high selectivity for monosilane synthesis.

As seen in Table 2, the main trichlorosilane disproportionation by-product is STC, which requires further processing. Regarding this, a bifunctional catalyst for TCS disproportionation and STC hydrogenation was proposed in this research. The activity of bifunctional catalysts based on transition metal chlorides was evaluated at a temperature of 160 °C. As seen from the results presented in Table 3, the bifunctional catalyst exhibits its activity both in the TCS disproportionation reaction and in the STC reduction reaction. The STC conversion decrease in catalytic systems modified with metal chloride particles in the series ReCl_3_ > NiCl_2_ > CoCl_2_ is consistent with the outcomes of Balandin’s multiplet theory of catalysis and the Newnes–Anderson model [36], [37]. In conclusion, there is the potential for further research on bifunctional catalysts, since they reduce the overall yield of STC, a toxic by-product of polycrystalline silicon production.

## 4. Materials and Methods

### 4.1. Reagents

Polyethylene glycol (PEG) having a molecular weight of 1500 was purchased from Sigma-Aldrich (Taufkirchen, Germany). KCl, HCl, hexane and toluene were purchased from Component-Reagent (Moscow, Russia). Helium (99.99999%) and argon (99.9995%) were purchased from Monitoring Ltd. (St-Petersburg, Russia). 1-Methylimidazole, 4(5)-methylimidazole and 3-chloropropyltriethoxysilane (CPTES) were purchased from Sigma-Aldrich (Taufkirchen, Germany). Imidazole and 2-methylimidazole were purchased from Tomsk Special Forces Reagent Facility Ltd. (Tomsk, Russia). Diethyl ether was purchased from Kuzbassorghim Ltd. (Kemerovo, Russia). Nickel chloride, cobalt chloride and rhenium chloride were purchased from Himreaktiv Ltd. (N. Novgorod, Russia). Trichlorosilane 99.998% and silicon tetrachloride (STC) were purchased from Firm HORST Ltd. (Dzerzhinsk, Russia). All reagents and solvents were used without further purification.

### 4.2. Preparation and Functionalization of Inorganic Support

SiO_2_ was synthesized using the sol–gel method in a glass jacketed flask. The process involved dissolving 8.4 g of PEG-1500 and 2.5 g of KCl in 60 mL of 2M HCl, followed by the addition of 8 mL of toluene or 8 mL of hexane. PEG with a molecular weight of 1500 was used as the silica sol flocculant because of its resistance to high temperatures during hydrothermal treatment. Using PEG-400 and PEG-700 led to degradation during hydrothermal treatment under these conditions. The mixture was stirred in a closed flask using a magnetic stirrer at 400 rpm for 2–3 h at 10 °C; then, TCS was added dropwise, and the reaction mixture was stirred for another 24 h. Then, hydrothermal treatment was performed by holding the mixture in an autoclave for 24 h at 100 °C. Next, the intermediate product, washed with deionized water through a Buchner funnel, underwent acid treatment in an autoclave with 60 mL of 2M HCl for 1–3 days at 100 °C. The filtered product was further washed with hexane in a Soxhlet apparatus for 24 h and then placed in a vacuum desiccator. Gradual heating to 100 °C with simultaneous pressure reduction to 10 mBar was carried out to prevent pore collapse. Calcination was performed to remove the surfactant matrix from the synthesized sample in a muffle furnace at 550 °C for 6 h in air.

**Figure 9:**
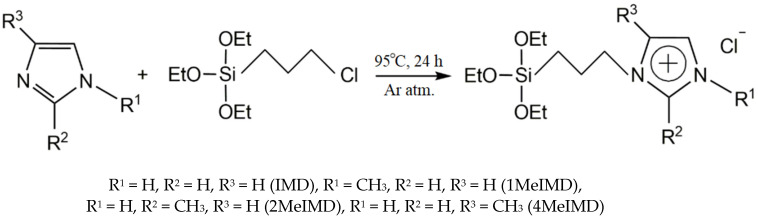
The scheme of the ionic liquids’ synthesis.

SiO_2_ functionalization was carried out using SILP technology (Figure 9). For this, 4 ionic liquids based on imidazole (IMD), 1-methylimidazole (1MeIMD), 2-methylimidazole (2MeIMD) and 4-methylimidazole (4MeIMD) were synthesized. They were mixed in a 1:1 molar ratio with 3-chloropropyl-triethoxysilane (CPTES) per 2 g of finished IL. IMD (1MeIMD, 2MeIMD or 4MeIMD) and CPTES were mixed in a 50 mL two-neck round-bottom flask filled with argon in the proportions shown in Table 4. The flask was placed in an oil bath with a magnetic stirrer, and a reflux condenser was connected. The synthesis was carried out under argon at 95 °C with stirring at 350 rpm for 24 h. Afterward, the product was cooled to room temperature and extracted twice with a double excess of diethyl ether. The resulting ILs were placed in a desiccator at room temperature under vacuum for 24 h. Table 1 shows the concentrations, densities and molar masses of the synthesized ILs.

**Figure 10:**
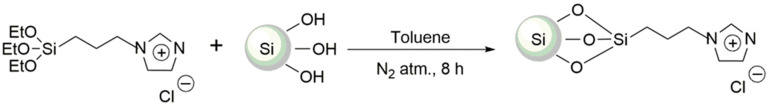
Immobilization of a porous inorganic support with synthesized ionic liquids.

The synthesized ILs were immobilized onto a porous support prepared by TCS (Figure 10). This involved adding 1 g of mesoporous silicon dioxide, 10 mL of toluene and 5 mL of IMD-CPTES IL into a 100 mL flask filled with an inert gas (nitrogen). The mixture was stirred for 8 h at room temperature, and then the product was washed with toluene in a Soxhlet apparatus for 24 h and placed in a vacuum to eliminate any solvent residues for 48 h.

**Figure 11:**
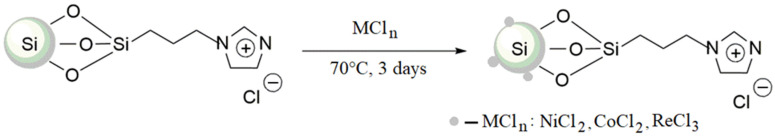
Scheme of the bifunctional catalysts’ synthesis.

For the bifunctional catalysts’ synthesis, the received SILPs were placed in saturated solutions of the corresponding metal chlorides (nickel, rhenium, cobalt) in ethyl alcohol, and the mixture was heated to 70 °C for 3 days (Figure 11). The resulting catalysts were filtered, washed with ethyl alcohol, and, to remove excess metal chloride, washed with acetone in a Soxhlet apparatus for 2 days. A notable indicator of the successful synthesis was a significant decrease in the chloride ion content after immobilization, determined by chloride ion titration with a standard solution of AgNO_3_. In addition, X-ray diffraction analysis confirmed the success of the synthesis.

### 4.3. Sample Characteristics

#### 4.3.1. Surface Area and Pore Distribution

The specific surface area and pore size distribution were determined from nitrogen absorption/desorption isotherms obtained using a SorbiMS META instrument (Russia) [40]. Two methods were used to determine the specific surface area—BET and STSA. The first one makes it possible to determine the specific surface area of micro- and mesopores in the relative pressure range of 0.06–0.2, using the monolayer model. The second one makes it possible to measure the specific surface area of meso- and macropores, as well as the external specific surface area, using a multilayer model [41], but not the specific surface area of micropores, since they are already filled in the relative pressure range of 0.2–0.5, which is used in this method. By employing the BJH method, the pore size distribution was determined from the adsorption isotherm, and the total pore volume was also determined by the same method within the relative partial pressure range (P/P0) of 0.95–0.98. Before the measurement began, degassing was carried out in the SorbiPrep sample preparation station in a flow of argon at a temperature of 250 °C for 1 h.

#### 4.3.2. SEM Analysis

The samples were analyzed for their morphological and visual characteristics using a scanning electron microscope (Merlin, Carl Zeiss, Germany). Samples were fixed to the sample holder using conductive carbon adhesive tape and coated with a 3 nm thick Au/Pd film using magnetron sputtering (Q150R S, Quorum, UK) to compensate for the charge induced by the electron beam. For microscopy, a secondary electron in-lens detector was used at an electron acceleration energy of 5–10 kV. The vacuum in the microscope chamber was about 10^–6^ mbar.

#### 4.3.3. Diffuse Reflectance Infrared Fourier Transform Spectroscopy (DRIFTS)

An IRTracer-100 Shimadzu (Tokyo, Japan) DRIFT spectrometer was used to characterize the silica samples. Thirty scans were averaged with a 4 cm^−1^ resolution over the range 4000–700 cm^−1^. To obtain the spectra, the samples were thoroughly ground in an agate mortar to a state that ensured a minimal scattering effect in the zinc selenide matrix. The components were mixed in a ratio that allowed the registration of granules’ absorption in the corresponding spectral range with minimal background absorption. To compare the obtained spectrum intensities, samples were prepared at a ratio of 1/9 by weight [12]. The mass of the powder under study for preparing the solution was 50 mg ± 1 μg.

### 4.4. Static Method for Evaluating Catalytic Activity

To evaluate the catalysts’ activity in the TCS disproportionation reaction, a static method was chosen, involving the holding of a mixture of TCS and helium in a thermostatically controlled reactor filled with the catalyst. The experiment was conducted under conditions of an excess catalyst related to trichlorosilane. A detailed experimental design is provided in [17]. The system was evacuated to a vacuum of 10^−5^ mbar at a temperature of 100 °C using a HiCube Eco 80 turbomolecular pump (Pfeiffer Vacuum, Germany) to remove trace amounts of moisture and air (degassing step to prevent hydrolysis). Next, the reactor was cooled to a reaction temperature of 80 °C, and TCS was introduced into the system until a pressure of 10 kPa was reached. The reactor was kept for 1 h, and then, when stationary conditions and thermodynamic equilibrium were established, the gas mixture from the reactor was analyzed using GCMS.

### 4.5. Product Analysis

The qualitative analysis of the main ex situ reaction products was conducted on a GCMS-QP2010Plus (Shimadzu, Japan) with a vacuum sample introduction system through an automatic injection valve (Valco Instruments Co Inc, USA). The reaction products were separated on an Agilent CP-7434 Select Silanes™ capillary column (30 m, 1.5 μm film) with a stationary phase based on cross-linked trifluoropropylmethylpolysiloxane at 323 K for 15 min; the carrier gas was helium. The reaction products were identified using the NIST-11 mass spectra database and the “CMS Real Time Analysis” program. According to the obtained data, the main products were SiH_4_ (*m*/*z* 30), SiH_3_Cl (*m*/*z* 64), SiH_2_Cl_2_ (*m*/*z* 99), SiHCl_3_ (*m*/*z* 133) and SiCl_4_ (*m*/*z* 170).

## 5. Conclusions

In this research, the catalytic systems’ synthesis was performed based on inorganic silica support with TCS as a precursor. By introducing additional solvents to the structure-forming agent PEG-1500, such as hexane and toluene, different cellular structures (hexagonal/spherical) could be formed, allowing the pore size and specific surface area to be regulated. A comparison of the contents of free silanol groups in silica samples from different precursors led to the conclusion that using TCS for the production of silica supports holds potential. A comparison of the catalytic activities of systems based on inorganic and polymer supports showed they are determined by the nature of the catalytic centers. Since it is known that with gas phase reactions, the availability of functional groups increases with the increase in specific surface area, as expected, a tendency for systems based on an inorganic support to increase the monosilane yield has appeared. Therefore, the IS/CPTES_4MeIMD^+^Cl^−^ catalyst exhibits the highest potential in the TCS disproportionation reaction; it showed the highest silane yield, 2.73%, and the maximum theoretical monosilane yield is 5.9%. The work proposes a bifunctional catalyst based on transition metal chlorides, demonstrating activity in two parallel reactions: TCS disproportionation and STC reduction.

## Figures and Tables

**Table 1:** Structural characteristics of the SiO_2_ samples obtained from TCS.

Sample	Solvent	BET, m^2^/g	STSA, m^2^/g	V_micro_, cm^3^/g	V_meso_, cm^3^/g	dV_i_/V_sum_, %
S1_T550	toluene	220.0 ± 4.2	94.3 ± 3.1	0.05	0.1	(101 nm) 24%
S2_T550	hexane	353.0 ± 2.1	195.0 ± 4.3	0.081	0.35	(57 nm) 37%
S3_HT_T550	toluene	301.2 ± 2.5	196.1 ± 3.6	0.08	0.3	(96 nm) 35%
S4_HT_T550	hexane	456.1 ± 2.1	245.1 ± 4.2	0.1	0.46	(54 nm) 26%
S5_HT_AHT100 _V100_T550	toluene	314.2 ± 2.1	201.0 ± 1.4	0.086	0.3	(29 nm) 27%
S6_HT_AHT100 _V100_T550	hexane	465.2 ± 5.2	334.1 ± 1.5	0.109	0.5	(29 nm) 36%

**Table 2:** Catalytic activity in the TCS disproportionation.

PS */IS **	MS *m*/*z* 30 (%)	MCS *m*/*z* 64 (%)	DCS *m*/*z* 99 (%)	TCS *m*/*z* 133 (%)	STC *m*/*z* 170 (%)
PS/IMD^+^Cl^−^	0.61	-	-	39.07	60.32
IS/CPTES_IMD^+^Cl^−^	0.76	-	-	37.22	62.02
PS/1MeIMD^+^Cl^−^	0.01	-	0.02	53.58	46.39
IS/CPTES_1MeIMD^+^Cl^−^	0.41	-	0.03	42.12	57.44
PS/2MeIMD^+^Cl^−^	0.04	0.10	-	48.91	50.95
IS/CPTES_2MeIMD^+^Cl^−^	0.82	-	-	43.47	55.71
PS/4MeIMD^+^Cl^−^	2.26	-	-	39.98	57.76
IS/CPTES_4MeIMD^+^Cl^−^	2.73	-	-	36.31	60.96

* PS—polymer support, ** IS—inorganic support.

**Table 3:** Dependence of the silane yield on various catalyst samples.

Sample	DCS Yield, mol %	STC Yield, mol %	STC Conversion, %
IS/CPTES_4MeIMD^+^Cl^−^	12.45	24.12	0
ReCl_3_∙IS/CPTES_4MeIMD^+^Cl^−^	12.20	18.57	23
NiCl_2_∙IS/CPTES_4MeIMD^+^Cl^−^	12.04	19.30	20
CoCl_2_∙IS/CPTES_4MeIMD^+^Cl^−^	11.86	20.02	17

**Table 4:** Properties of synthesized ILs.

	IMD	1MeIMD	2MeIMD	4MeIMD	CPTES
molar mass, g/mol	68.1	81.1	82.1	82.1	240.8
density, g/mL	1.23	1.03	-	1.02	1
V, mL	0.355	0.494	-	0.499	1.491
m, g	0.431	0.509	0.509	0.509	1.491

## Data Availability

Data are contained within the article.
